# Global Transcriptomic Analysis Reveals the Mechanism of *Phelipanche aegyptiaca* Seed Germination

**DOI:** 10.3390/ijms17071139

**Published:** 2016-07-15

**Authors:** Zhaoqun Yao, Fang Tian, Xiaolei Cao, Ying Xu, Meixiu Chen, Benchun Xiang, Sifeng Zhao

**Affiliations:** Key Laboratory at Universities of Xinjiang Uygur Autonomous Region for Oasis Agricultural Pest Management and Plant Protection Resource Utilization, Shihezi University, Shihezi 832003, China; yaozhaoqun@sina.com (Z.Y.); shztianfang@sina.com (F.T.); tulanduoleileicx@sina.com (X.C.); xyella@163.com (Y.X.); cmx19930519@sina.com (M.C.); xbc@shzu.edu.cn (B.X.)

**Keywords:** *Phelipanche aegyptiaca*, seed germination, ABA, strigolactones, GR24, transcriptome sequencing

## Abstract

*Phelipanche aegyptiaca* is one of the most destructive root parasitic plants of Orobanchaceae. This plant has significant impacts on crop yields worldwide. Conditioned and host root stimulants, in particular, strigolactones, are needed for unique seed germination. However, no extensive study on this phenomenon has been conducted because of insufficient genomic information. Deep RNA sequencing, including de novo assembly and functional annotation was performed on *P. aegyptiaca* germinating seeds. The assembled transcriptome was used to analyze transcriptional dynamics during seed germination. Key gene categories involved were identified. A total of 274,964 transcripts were determined, and 53,921 unigenes were annotated according to the NR, GO, COG, KOG, and KEGG databases. Overall, 5324 differentially expressed genes among dormant, conditioned, and GR24-treated seeds were identified. GO and KEGG enrichment analyses demonstrated numerous DEGs related to DNA, RNA, and protein repair and biosynthesis, as well as carbohydrate and energy metabolism. Moreover, ABA and ethylene were found to play important roles in this process. GR24 application resulted in dramatic changes in ABA and ethylene-associated genes. Fluridone, a carotenoid biosynthesis inhibitor, alone could induce *P. aegyptiaca* seed germination. In addition, conditioning was probably not the indispensable stage for *P. aegyptiaca*, because the transcript level variation of MAX2 and KAI2 genes (relate to strigolactone signaling) was not up-regulated by conditioning treatment.

## 1. Introduction

A total of 4100 plant species in approximately 19 families of flowering plants have evolved to parasitize other plants. These plants are widely distributed in Africa, Europe, Asia, America, and Australia. Haustoria are used by these plants to obtain water and nutrients from other living plants or hosts [1,2]. Parasitic weeds cause serious agricultural problems, resulting in remarkable crop losses in many regions worldwide. About $1.3 to 2.6 billion of annual food crop losses are caused by broomrape infestation [3]. *Phelipanche* spp. are holoparasites that lack chlorophyll. They parasitize more-temperate climate crops, such as sunflower, tomato, potato, tobacco, carrot, clovers, cucumber, rapeseed and legumes [4]. *Orobanche cumana* has damaged 20,000 ha of farmland in Greece and China, with estimated yield losses of 60% in Greece and 20%–50% in China. In 1994, *Phelipanche aegyptiaca* had extensive infestations of muskmelon and watermelon, leading to 20%–70% yield losses in Xinjiang Province, China [5]. The life cycle of *Phelipanche* spp. has a number of mechanisms that coordinate the life cycles of parasites to that of their host. The main steps in the life cycle are conditioning of seeds, germination under stimulants secreted by hosts, adhesion and formation of appressorium, penetration through host tissues, formation of haustorium to connect the host vascular tissues, development of a tubercle and apex, stem growth and emergence, and flowering and seed production [6,7,8]. The seeds of *Orobanche* spp. contain only little reserves. These seeds can survive for a few days only after germination unless they reach a host root to establish a xylem connection.

The *Orobanche* spp. parasitic strategy generally succeeds by coordinating early developmental stages with chemical signals from hosts. An important step in the life cycle of *Orobanche* spp. is their germination at the right place and time, enabling them to establish the connection they require to survive. *Orobanche* spp. usually use so-called germination stimulants secreted by roots of their hosts. To date, three different types of compounds, namely, dihydroquinones (dihydrosorgoleone), sesquiterpene lactones, and strigolactones (SLs), have been identified as chemical signals or germination stimulants for *Striga* spp. and *Orobanche* spp. Among these germination stimulants, SLs are the most active in inducing germination at 10^−7^ to 10^−15^ mol/L [9,10]. SLs are new plant hormones that control shoot branching, root architecture, cambial growth, and senescence [11,12]. SLs are synthesized from carlactone, which is derived from all-trans β-carotene via the action of an isomerase (D27) and two carotenoid cleavage dioxygenases (CCD7 and CCD8). Then, further ring closures and functionalizations involves in members of the CYP711 family (MAX1). Once synthesized, SLs may be transported by PhPDR1, a member of the ABC family within the plant and in the rhizosphere. Finally, MAX2 interacts with D14/KAI2 in an SLs-dependent manner, and this leads to SL ubiquitylation dependent degradation of D53 by the SCF^MAX2^ complex [10,11]. Further, these hormones also serve as extra organismal signals in soil that recruit symbioses with arbuscular mycorrhizal fungi and trigger Orobanchaceae plant seed germination [13].

*Orobanche* spp. exerts the greatest damage prior to their emergence, and the majority of field loss may occur before diagnosis of infection. Numerous physical, cultural, chemical, and biological approaches have been explored against root parasites. However, none of these techniques are effective and provide economical results [14,15,16,17,18]. SLs are regarded as potential new strategies to control *Orobanche* spp. [19]. The tomato SL-deficient mutant (*SL-ORT1*) displays resistance to *P. aegyptiaca* because of the inability of *SL-ORT1* roots to produce and secrete natural germination stimulants (SLs) to the rhizosphere. Silencing of the tomato *MAX3/CCD7* gene, which is the critical gene for SL production, reduces the number of *P. ramose* infection [20,21]. AM symbiosis in tomato also reduces SL production and *P. ramosa* infection [22]. In 2016, it was successful to reduce *O. ramosa* in tobacco fields by using SL analogues via suicidal germination approach [23]. However, genomic and molecular resources for *Phelipanche* are limited [24], and the mechanism of SLs inducing *Phelipanche* seed germination remains unclear.

Transcriptome and proteome technology can facilitate the understanding of the molecular basis of complex developmental processes [25]. De novo assembly and characterization of the transcriptome of *Cuscuta pentagona* and three parasites of Orobanchaceae have uncovered genes associated with plant parasitism [26,27,28]. Transcriptome sequencing successfully provided new insight into seed germination processes in *Arabidopsis*, wheat, and cotton [29,30,31]. In the present study, dormant, conditioned, and GR24 (a synthetic analog of SL)-treated *P. aegyptiaca* seeds were used to assemble and annotate a reference transcriptome. The transcriptome data were then used to analyze different expressions of genes during different seed germination stages. Finally, the role of plant hormones in *P. aegyptiaca* seed germination was investigated by physiological tests using differentially expressed genes (DEGs) and Kyoto Encyclopedia of Genes and Genomes (KEGG) pathway analysis.

## 2. Results

### 2.1. De Novo Assembly of P. aegyptiaca Transcriptome

**Figure 1:**
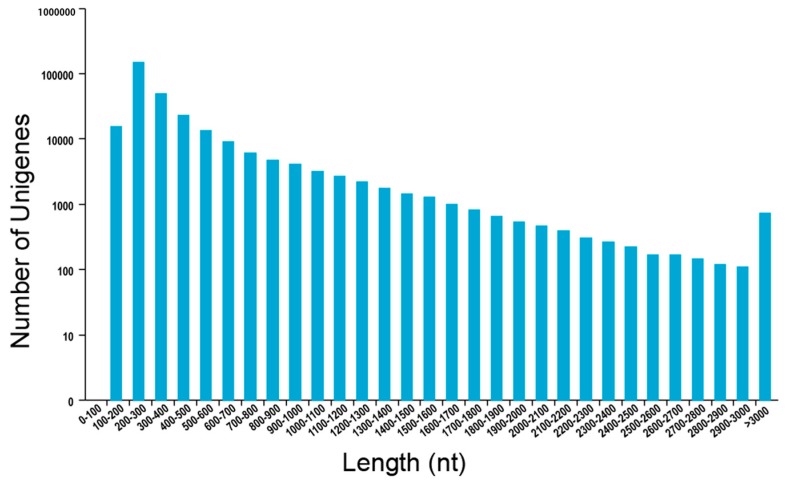
Length distribution of Trinity assembly for unigenes of *Phelipanche aegyptiaca*.

RNA-sequencing (RNA-seq) library was prepared from dormant, conditioned, and GR24-treated seeds and sequenced using the Hiseq 2500 platform. A total of 78,540,698 reads were obtained. The percentage of Q30 base in all samples was over 88.36%. Illumina adapters were trimmed, and low-quality bases were filtered. Trinity software package [32] was used to assemble clean data. A total of 274,964 transcripts (longer than 200 bp) were found. The average length of N50 of transcripts was 1066 bp, and the evaluated number of unigenes was 94,419 (Table 1 and Figure 1).

### 2.2. Transcriptome Functional Annotation

Various approaches were used to acquire comprehensive information on the assembled transcriptomes. First, unigenes were used for homology searches against the non-redundant (NR), Gene Ontology (GO), Cluster of Orthologous Groups of proteins (COG), KOG, and KEGG databases using the BLASTX algorithm, with an *E*-value ≤ 10^−5^. Subsequently, amino acid sequences from predicted unigenes were aligned against the Pfam database with an *E*-value ≤ 10^−6^ using HMMER software. Thus, 53,921 unigenes were annotated. However, numerous unigenes (40,498) remained unannotated. Annotation statistics of BLASTX hits and domain hits are summarized in Table S1.

**Figure 2:**
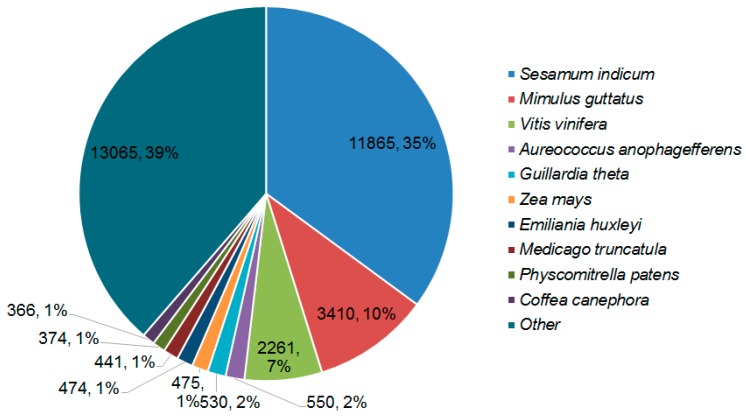
Hit species distribution of BLASTX matches of *P. aegyptiaca* transcriptome. Number and proportion of *P. aegyptiaca* transcripts with similarity to sequences from the NR database.

BLASTX against the NR database provided insight into the taxonomic distribution of the transcripts. High mapping rates of contigs to the protein databases suggested that most of the contigs could be translated into proteins. BLASTX hits and top hits in terms of the total numbers of hits of all transcripts were mostly observed in *Sesamum indicum* (11,865 hits), *Mimulus guttatus* (3410 hits), and *Vitis vinifera* (2261 hits) (Figure 2).

**Figure 3:**
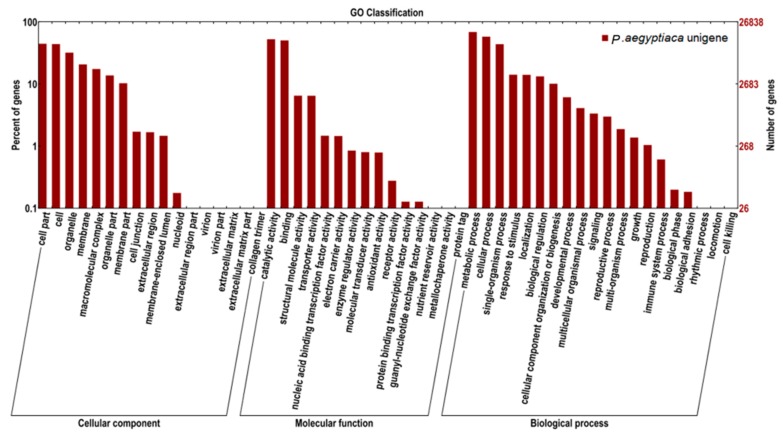
GO category distribution of *P. aegyptiaca* transcripts among level 1 GO categories: Biological process, molecular function, and cellular component.

GO is an international classification system for standardized vocabulary, which is used in the comprehensive description of functions of uncharacterized genes. The three main independent GO categories are biological processes, molecular functions, and cellular components. Our annotated unigenes were assigned to GO terms for functional classification. A total of 21,851 (27.3%), 21,961 (27.5%), and 23,287 (29.2%) unigenes were annotated in the biological process, molecular function, and cellular component categories, respectively (Figure 3). The majority of unigenes under cellular component were involved in cells (23.42%), cell parts (23.68%), and organelles (17.05%). Approximately 43.14% and 41.33% of unigenes within the molecular function category were clustered in catalytic activity and binding, respectively. Sub-categories metabolic (28.19%), cellular (23.76%), and single-organism (17.93%) processes were the major sub-categories of biological process.

### 2.3. qRT-PCR Validation

**Figure 4:**
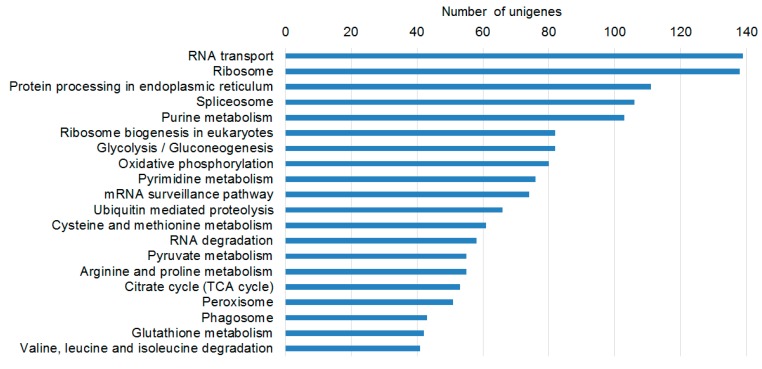
Top 20 KEGG pathways in *P. aegyptiaca* germinating seeds according to the numbers of DEGs. This Figure shows the KEGG metabolic pathways of plants that were well represented by unique sequences of *P. aegyptiaca*.

**Figure 5:**
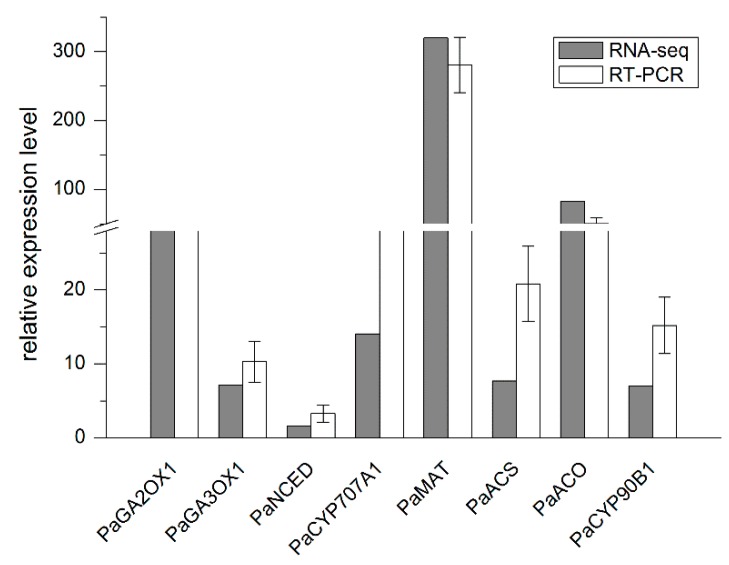
Validation of hormone-related genes at the GR24 treatment stage by qRT-PCR. Fold changes were originally estimated in the RNA-seq experiment (brown bar) and validated by qRT-PCR (white bar). The results are the average of three biological replicates. Abbreviations of genes: PaGA2OX1, gibberellin 2-beta-dioxygenase 1; PaGA3OX1, gibberellin 3-beta-dioxygenase 1; PaNCED, 9-*cis*-epoxycarotenoid dioxygenase; PaCYP707A1, ABA 8′-hydroxylase 1; PaMAT, methionine adenosyltransferase; PaACS, amino cyclopropanecarboxylate synthase; PaACO, aminocyclopropanecarboxylate oxidase; PaCYP90B1, cytochrome P450 90B1.

We also used RNA samples isolated for RNA sequencing to perform qRT-PCR analysis (Figures S1 and S2). The 12 randomly chosen genes and 8 genes relating to plant hormone (GA, ABA, ethylene and BR) biosynthesis were used to qRT-PCR validation. From the supplemental Figure S2, the 12 gene dynamic changes of qPCR and the RNA-seq results were consistent. Furthermore, expression levels of the 8 plant hormone genes are also supported by the changes of each hormone content change (Figure 4 and Figure 5). These results suggested the applicability of RNA-Seq to *Phelipanche* transcriptome analysis is an accurate and reliable way to find DEGs during *P. aegyptiaca* seed germination.

### 2.4. Comparative Analysis of Differential Expression during Germination

We used RPKM (reads aligned to gene per kilobase of exon per million mapped reads) to estimate gene expression values to better understand the dynamic performance among the three different transcriptomes during seed germination. False discovery rate (FDR) <0.01 and fold change ≥2 were used as cutoff values to identify differentially regulated transcripts. A total of 5324 DEGs among dormant, conditioned, and GR24-treatedseeds were identified. A total of 633 and 61 unigenes were up-regulated and down-regulated in dormant seeds, respectively, compared with conditioned seeds. A total of 3764 unigenes were up-regulated and 866 unigenes were down-regulated between conditioned and GR24-treated seeds (Figure S3).

**Figure 6:**
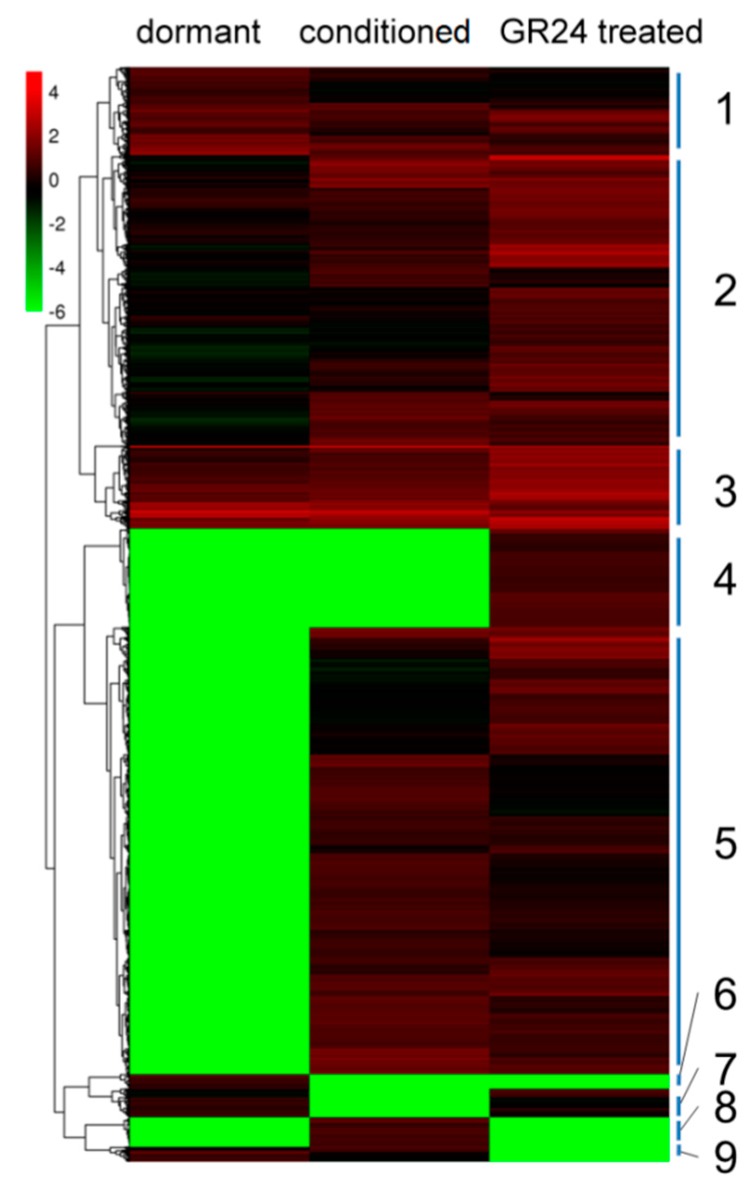
Heat map from hierarchical clustering of differentially expressed genes during *P. aegyptiaca* seed germination. Expression changes and cluster analysis of 5324 genes that were differentially expressed between any two of the three samples. Each row represents a differentially expressed gene (DEG), whereas each column represents a sample. Changes in expression levels are shown in color scales with saturation at >2.0-fold changes. Green and red color gradients indicate a decrease and increase in transcript abundance, respectively.

Based on the DEGs among the three samples, clustering analysis was performed via MeV edition 4.90 using the algorithm of “hierarchical clustering” (Figure 6). Groups of unigenes with similar expression patterns under different stages were measured in nine clusters. Gradual changes in the gene expression patterns of clusters 1, 2, and 3 varied, and nearly half of DEGs belonged to these three clusters. DEGs of cluster 4 were not active until GR24 application. These genes were probably regulated by GR24, and play crucial roles in the last germination stage. Cluster 5 contained 1840 DEGs that were up-regulated during seed germination. These genes were evidently not only involved in plant hormones but also related to many metabolic pathways. However, the roles of these genes during *P. aegyptiaca* germination have not been elucidated. DEGs in cluster 6 were examined in dormant seeds, so these genes inhibited *P. aegyptiaca* seed germination, contrary to the expression patterns of genes of clusters 7 and 8. Thus, cluster 7 genes probably inhibited germination, in contrast to the promotion of germination by cluster 8 genes. The inhibition or promotion of the genes in clusters 7 and 8 was released or induced by conditioning, respectively. DEGs in the last group, cluster 9, were down-regulated gradually in chronological order. These genes played more important roles in dry seed than during seed germination.

Furthermore, to determine the biological significance of the DEGs, GO terms enrichment analysis of the total DEGs was also carried out (*p* ≤ 0.05). As shown in Figure S4, the biological processes of translation (GO:0006412), response to salt stress (GO:0009651), oxidation-reduction process (GO:0055114) were involved in 221, 44 and 43 DEGs, respectively, indicating mRNA translation, stress resistance and energy metabolism played a vital role in conditioned phase. Between the conditioned and GR24 treatment stage, 151, 119, 47 and 43 DEGs were enriched in the oxidation-reduction process (GO:0055114), translation (GO:0006412), metabolic process (GO:0008152), and protein phosphorylation (GO:0006468), respectively. This suggested energy metabolism and mRNA translation still play dominate roles, and protein and other metabolic process were carried out.

### 2.5. Gene Change during P. aegyptiaca Seed Germination

Removal of the seed coat permeability barrier, leaching of germination inhibitors, facilitating the access of stimulants to their putative cellular target, biosynthesis of plant hormones, and formation or activation of receptors for germination stimulants have been inferred to occur during conditioning [33,34]. DEGs between dormant and conditioned seeds showed the two biggest group of genes involved from DNA to protein repair and biosynthesis, as well as carbohydrate and energy metabolism (Figure 5). Tested *P. aegyptiaca* seeds and endogenous germination inhibitors have not been proven to inhibit the germination of this plant. We found that the gibberellin 20 oxidase 1 (GA20OX1) transcript was up-regulated and GA3 levels increased during conditioning. In addition, the 3-epi-6-deoxocathasterone 23-monooxygenase (CYP90C1) transcript was up-regulated. This cytochrome P450 enzyme plays an important role in early C-22 oxidation of brassinosteroid (BR) biosynthesis. The expression levels of the two genes were supported by quantitative real-time polymerase chain reaction (qRT-PCR) (Figure 6).

The second phase started after stimulation, during which sharp transition in *Phelipanche* seeds occurred [35]. Dominant DEGs were associated with DNA, RNA, protein, carbohydrate, and energy metabolism. More genes and higher expression levels were found during this phase. Additionally, genes involved in plant hormone biosynthesis and metabolism were dramatically up-regulated. Gibberellin 2-beta-dioxygenase 1 (GA2OX1) gene and gibberellin 3-beta-dioxygenase 1 (GA3OX1) transcript were up-regulated significantly, and GA3 levels increased. Cytochrome P450 90B1 (CYP90B1) gene, which encodes a BR biosynthesis enzyme, was activated dramatically, and the amount of BRs increased. The tendencies of the two phytohormones levels were apparently similar to those during the conditioning stage. Numerous studies have proven that ethylene can promote seed germination. Our data showed that in methionine adenosyltransferase (MAT), aminocyclopropanecarboxylate synthase (ACS), and aminocyclopropanecarboxylate oxidase (ACO), which are three key enzymes of ethylene biosynthesis, corresponding genes were up-regulated significantly after GR24 treatment (Figure 6). The 9-*cis*-epoxycarotenoid dioxygenase transcript, which plays a crucial role in abscisic acid (ABA) biosynthesis, was dramatically down-regulated. In addition, in ABA 8′-hydroxylase 1 (CYP707A1), which catalyzes hydroxylation at the C-8′ position of ABA, the transcript was up-regulated. These results coincided with the qRT-PCR results (Figure 6) and ABA level reduction.

As Kusumoto reported, receptors for germination stimulants are perhaps formed or activated during conditioning [34]. Transcriptome results showed that the F-box protein MAX2 expression level remained high in the whole germination process, which is associate with perception of SLs [36]. Furhter, two α/β-hydrolase KAI2-like genes were identified in our results, the expression of one was high, and the other expression level even down-regulated during the germination process (Figure S5). These results demonstrated that the SL receptor complex was not only active during conditioning or after GR24 application, but it was more likely active all the time.

### 2.6. Endogenous Hormone Levels and Germination Test

**Figure 7:**
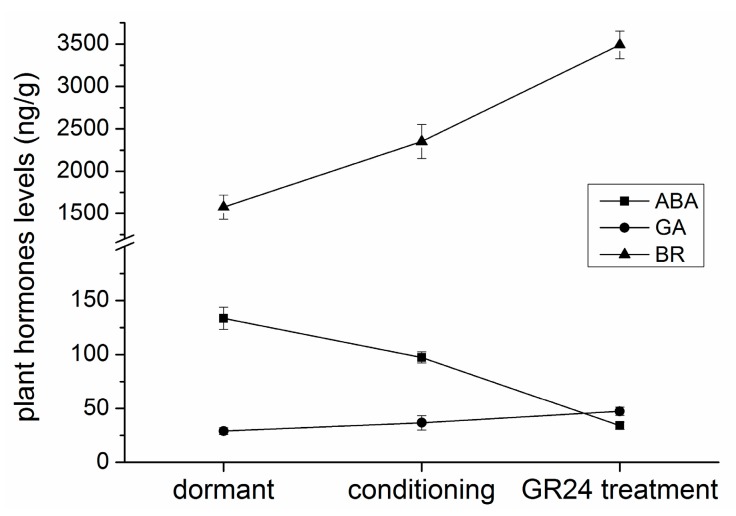
Time course of ABA, GA3, and brassinosteroid (BR) contents in *P. aegyptiaca* seed germination. Seeds were conditioned in water at 25 °C in the dark for 7 days and then conditioned in GR24 (10^−6^ mol/L) at 25 °C for 1 day.

Changes in the endogenous plant hormone levels were tested to elucidate the roles of hormones. GA3, BRs, and ethylene levels continuously increased, similar to those in previous reports on seed germination. Interestingly, GA3 and BR levels gradually increased, contrary to the increase in ethylene level after GR24 application. An initial slight decrease in the ABA level was followed by a significant reduction after GR24 treatment (Figure 7).

A germination experiment was designed to explore the key changes in hormone levels for *P. aegyptiaca* seed germination. The results showed that individual application of the four hormones failed to induce *P. aegyptiaca* seed germination. However, fluridone, the inhibitor of carotenoid biosynthesis, promoted seed germination without GR24 application. GA3, BRs, and ethephon (it can be converted into ethylene at pH < 7) significantly help fluridone increase germination rates. GA3, fluridone, or GA3, as well as ethephon and fluridone, induced seed germination without GR24 application (Table 2 and Figure S6). These results were probably due to the enhanced GA/ABA ratio caused by exogenous fluridone, as well as the GA3 and ethylene-induced changes in the GA/ABA ratio value by adjusting the ABA content. Therefore, the change in ABA level may be the key factor in *P. aegyptiaca* seed germination, which is controlled by GR24.

## 3. Discussion

Transcriptome sequencing was used to identify the role of GR24 in *P. aegyptiaca* seed germination. Three developmental stages from dormant to GR24-treated seeds were selected. Several key genes and pathways were determined. Protein biosynthesis and metabolism, as well as energy metabolism, were dominant in *P. aegyptiaca* seed germination. Additionally, many genes were involved in plant hormone biosynthesis and transduction.

### 3.1. Transcriptome Assembly and Annotation

RNA-seq reads of dormant, conditioned, and GR24-treated seeds were used to assemble a reliable transcriptome. However, only 61% of the filtered transcripts were annotated. This result was similar to many other de novo-assembled plant transcriptomes, such as *Capsicum frutescens*, *Nicotiana benthamiana*, and *Cuscuta pentagona* [27,37,38]. We found that 35% hits belonged to *S. indicum*, the genome of which was sequenced in 2014 [39]. Although the taxonomic position of *Phelipanche* and *Striga* is closer compared with *Sesamum* and *Mimulus*, belonging to Lamiales, the two top hits results are *S. indicum* and *M. guttatus*. This resulted from a lack of *Striga* genome data.

### 3.2. DEGs Associated with Broomrape Seed Germination

#### 3.2.1. DNA, RNA, and Protein

Numerous stored mRNAs, such as for seed reserve synthesis, late embryogenesis abundant proteins, protein synthesis, and degradation in mature dry seeds, are necessary for germination [40,41,42]. Degradation of stored mRNAs is a prerequisite to germination [43,44]. In our study, Trf4/5, CONT3, and PABP1 transcripts related to RNA degradation were up-regulated in the conditioned phase. By contrast, CNOT3 and DDX6 transcripts related to RNA degradation were down-regulated after GR24 application. Stored proteins were used to restart cellular activity, followed by mobilization of the stored mRNA pool [42].

Hunt and Macovei reported the DNA repair and replication in *Arabidopsis* and *Medicagotruncatula* seed germination [45,46]. We observed that xerodermapigmentosum complementation group C (XPC) and the HR23B genes of the DNA repair XPC complex were up-regulated. Replication protein A, which is a DNA replication protein, and ligase Lig I genes were up-regulated after GR24 treatment. Poly (ADP-ribose) polymerases (PARPs) were implicated in DNA repair in *Arabidopsis* [45]. We found that PARP transcripts were very high in dormant and conditioned seeds, but their expression was dramatically down-regulated after GR24 treatment. Thus, DNA repair may have been completed during the conditioning stage.

De novo RNA synthesis started after DNA repair and replication. α-amanitin, which is a highly potent and specific inhibitor of DNA-dependent RNA polymerase II, strongly affected the speed and uniformity of *Arabidopsis* seed germination [47]. Our data also showed that the DNA-dependent RNA polymeraseII transcripts were up-regulated during the entire germination period. Pentatricopeptide repeat proteins genes were up-regulated, which also participate in RNA splicing, cleavage, editing, stability, and translation [48].

De novo protein synthesis during early seed germination was indispensable. As Rajjou demonstrated, the germination of *Arabidopsis* seeds is entirely blocked in the presence of cycloheximide, a translation inhibitor [47]. However, free amino acids in dry seeds are not sufficient for protein synthesis during germination [49]. Therefore, storage protein degradation is an optimal way to solve this problem. The protein L-isoaspartyl-O-methyltransferase (PIMT) repair pathway facilitates the active elimination of deleterious protein products during germination [50]. Although we did not observe PIMT transcript activity, the two main protein degradation KEGG pathways, namely, proteasome- and ubiquitin-mediated proteolysis, were activated during the conditioning stage. Furthermore, majority of transcripts in both pathways were down-regulated in the presence of GR24.

#### 3.2.2. Energy Metabolism

Energy is supplied from cellular respiration, including glycolysis of carbohydrates and β-oxidation of fatty acid, even amino acids, during seed germination. All biochemical reaction products feed into the tricarboxylic acid (TCA) cycle and provide power for mitochondrial electron transport chain to produce ATP [51].

The two main pathways for carbohydrate metabolism are the oxidative pentose phosphate pathway (OPPP) and glycolysis (Figure S7). Glucose-6-phosphate dehydrogenase (G6PDH) plays a key role in OPPP, and its expression level was stable during germination. By contrast, two key enzymes in the glycolysis pathway, namely, 6-phosphofructokinase (PFK) and pyruvate kinase (PK) transcripts, were up-regulated during the conditioning stage. After treatment with GR24, α-amylase and PFK gene expression levels was down-regulated. This phenomenon suggested that carbohydrates were depleted after conditioned treatment.

Thus, fatty acid or protein acted as an energy supplement in the next germination phase (Figure S7). Many lipoxygenases and fatty acyl-CoA synthetase, acyl-CoA dehydrogenase, enoyl-CoA hydratase, β-hydroxyacyl-CoA dehydrogenase, and acetyl-CoA acetyltransferase transcripts involved in fatty acid β-oxidation were active. Isocitratelyase (ICL), which is a key enzyme in the glyoxylate cycle, and its transcript was active. TAG provides energy to the embryo before the initiation of photosynthesis [52]. Although our results demonstrated that two crucial enzymes, namely, glycerol-3-phosphate dehydrogenase (G3PDHc) and triacylglycerol (TAG) lipase genes, were not activated, glycerol kinase expression increased after GR24 application. Therefore, fatty acid was the energy donor processed through both the glyoxylate cycle and glycerol shunt pathway (Figure S7).

Aspartate and alanine (AspAT) and aminotransferases (AlaAT) genes were activated during imbibition and inferred to participate in respiratory pathways [51]. Our data illustrated that the corresponding genes of both enzymes were not activated during the conditioning phase. However, only the AspAT transcript was activated after GR24 application. The activity and transcript levels of glutamate decarboxylase (GAD), which is the entry enzyme of the aminobutyrate (GABA) shunt, increase in germinating seeds [43,53]. GAD transcript expression level in *P. aegyptiaca* was high at the conditioning stage and upon GR24 application. Oxaloacetate is another product of aspartate. This product can feed into the TCA cycle either by transforming to PEP under PEPCK or by conversion to malate by cytosolic malate dehydrogenase (MDHc) (Figure S7). Our result showed that the PEPCK gene was activated during the whole developmental process, but MDHc transcripts were down-regulated after GR24 treatment. Thus, further studies are necessary to determine the manner by which the two pathways of amino acids supplying energy cooperate with each other during *P. aegyptiaca* seed germination at different stages.

Pyruvate, as the energy metabolism hub, feeds acetyl-CoA to the TCA cycle through pyruvate dehydrogenase complex (PDC) and alcohol dehydrogenase (ADH) or lactate dehydrogenase (LDH) under aerobic the condition, contributing to the transformation of pyruvate into ethanol or lactate, respectively. PDC-related genes were activated after imbibition and GR24 application. The genes of the entire coded TCA cycle enzymes, including malate dehydrogenase in mitochondria, citrate synthase, aconitatehydratase, isocitrate dehydrogenase, oxoglutarate dehydrogenase complex, succinate-CoA ligase, succinate dehydrogenase (SDH), and fumaratehydratase transcripts were up-regulated (Figure S7). The activity of these genes also highly increased during the early germination stage in many plants [51]. However, GR24 treatment resulted in the down-regulation of SDH and fumaratehydratase transcripts. This phenomenon needs further investigation. Additionally, the fermentation-related transcript, pyruvate dehydrogenase transcript, was up-regulated, contrary to LDH.

#### 3.2.3. Phytohormone Activity

ABA inhibits both embryo growth and endosperm rupture to establish seed dormancy and inhibit germination. Nevertheless, GAs counteracts ABA responses and promotes seed germination [54]. Several studies have shown that ABA levels decrease during *P. aegyptiaca* seed conditioning [55]. ABA biosynthesis inhibitors, namely, fluridone and norflurazon, can promote dormancy release and germination [56,57]. Application of exogenous ABA inhibits *P. ramosa* seed germination [57]. In this study, changes in NCED and CYP707A1 transcripts coincided with the change in ABA level (Figure 7) and results from previous studies [57,58,59]. A recent study on *P. ramosa* seeds demonstrated that the seed response to SL is controlled by ABA-independent DNA methylation [60]. Thus, exogenous ABA biosynthesis inhibitor fluridone was examined and found to induce *P. aegyptiaca* seed germination without GR24. Although a large number of previous studies showed SLs act in a positive role with respect to seed germination, as ABA and SLs share a biosynthesis pathway, so this phenomenon resulting from the effect of ABA or SLs needs further discussion. In many studies on *Striga* and *Orobanche*, fluridone alone was not able to induce their seed germination [34,55,61,62,63], but our results showed that application of fluridone without GR24 could induce seed germination. We speculate the different results occurred because the *Phelipanche* seed used in studies was different, and this exciting phenomenon needs more investigation.

Several studies provided evidence that GA synthesis occurs during conditioning [64]. In our experiment, the GA20OX1 transcript was up-regulated after conditioned treatment and GR24 induced GA2OX1 and GA3OX1 transcript up-regulation. Some studies showed that application of GA biosynthesis inhibitors (e.g., uniconazole) during seed conditioning inhibits the subsequent germination of *Striga hermonthica*, *P. ramosa*, *P. aegyptiaca*, and *O. minor* in response to GR24 [57,62,65]. BR application after conditioning increased the rate of seed germination induced by stimulants [56]. CYP90C1and CYP90B1 transcripts were up-regulated during different germination stages. Although BR level increased during the whole process, BRs were inferred to alter the balance of other endogenous phytohormones, such as indole-3-acetic acid (IAA), ABA, GAs, and ethylene rather than acting directly [66]. However, we found that exogenous GA3 or BR promoted *P. aegyptiaca* seed response to GR24, but individual application of these two hormones failed to induce seed germination.

Ethylene, which is another plant hormone, also plays an important role in seed germination by promoting the weakening of the micropylar endosperm [67,68]. This hormone can neutralize many of the negative functions of ABA during germination [69,70,71]. Ethylene, ethephon, or aminocyclopropanecarboxylate (ACC) stimulates seed germination in numerous species, including several parasitic plants, such as *P. ramosa* [72] and some *Striga* species [73,74]. The results showed that methionine adenosyltransferase (MAT), ACS, and ACO expression levels were induced by GR24. This result was supported by Zehhar’s study, in which ethylene synthesis was required for the induction of *P. ramosa* seed germination by GR24 [57]. However, we failed to quantify ethylene accurately; low endogenous ethylene was probably released into the medium. However, ethylene down-regulated ABA accumulation by both inhibiting its synthesis and promoting its inactivation, as well as negatively regulated ABA signaling [75]. Thus, indirect degradation by controlling ethylene requires further examination.

The ABA biosynthesis pathway was inhibited, and its degradation was promoted by GR24. This is consistent with the research on CYP707A in the seed germination of *P. ramosa* [58,60]. By contrast, the ethylene biosynthesis pathway was activated. Therefore, the bottleneck of *P. aegyptiaca* seed germination was endogenous ethylene and the ABA level, which were probably controlled by SLs.

SLs carry out their physiological functions via a specific reception system similar to other plant hormones. SLs bind to the D14/MAX2/SCF complex, and then lead to SMAX1, a D53 homolog, degradation, finally acting on seed germination, and this model is widely accepted [11]. However, recently, it was reported that the α/β-hydrolase KAI2 gene (D14-like, also known as HTL) plays a central role in the perception of SLs [36,76]; this gene was thought to be the key gene in reception of the strigolactone-analogous compound karrikin [11], originally found in forest-fire smoke. The transcriptome and qRT-PCR analysis of MAX2, D14, and two KAI2-like genes illustrated their expression levels are stable during seed germination. We only found two KAI2-like genes in our data; however, there are 11 KAI2 genes in *S. hermonthica* [76]. According to the results, we conservatively speculate the transcripts of partial components of the SL perception complex existed prior to conditioning.

## 4. Materials and Methods

### 4.1. Sample Preparation

Mature seeds of *P. aegyptiaca* were collected from *Cucumis melo* L. in the Hami area of the Xinjiang Uygur Autonomous Region, China, in 2012. Dormant, conditioned, and GR24-treated seeds were used in this study. All seeds were surface-sterilized by 75% ethanol for 1 min and 1% sodium hypochlorite for 20 min followed by three rinses in sterile distilled water [77]. Subsequently, 10 g of seeds were frozen in liquid nitrogen as the dormant seed sample and then stored at −80 °C until RNA was extracted. The conditioned seed sample was treated as follows: 10 g of sterilized seeds were spread on sterilized glass fiber filter paper moistened with sterile distilled water. The seeds were placed into 10 cm Petri dishes and incubated in the dark at 25 °C/20 °C for 7 days [60]. The seeds were then frozen in liquid nitrogen and stored at −80 °C until RNA was extracted. The GR24-treated seed sample consisted of 10 g of conditioned seeds induced by applying GR24 at 10−6 mol/L (0.1% acetone, *v*/*v*) for 1 day, frozen in liquid nitrogen, and stored at −80 °C until RNA was extracted [78].

### 4.2. RNA-seq Library Preparation and Sequencing

Total RNA of each sample was isolated using Trizol Reagent (Invitrogen, Gaithersburg, MD, USA) according to the manufacturer’s instructions. RNA quality was characterized by agarose gel electrophoresis and spectrophotometry. High-quality RNA, with 28S:18S of more than 1.5 and absorbance 260/280 ratio between 1.8 and 2.2, was used for library construction and sequencing. Illumina HiSeq2500 library was constructed according to the manufacturer’s instructions (Illumina, San Diego, CA, USA). Magnetic beads with oligo (dT) (Thermo Scientific, Waltham, MA, USA) were used to enrich the mRNA from total RNA. mRNA was randomly cleaved by adding fragmentation buffer. The fragments were used to synthesize first-strand cDNA with random hexamers. After first-strand cDNA was synthesized, the buffer, dNTPs, RNase H, and DNA polymerase I were added to synthesize double-stranded cDNA (dscDNA), which was purified using AMPure XP beads. The purified dscDNA was end repaired, added to an A-tail, and linked with sequencing adapters [79]. AMPureXP beads (Beckman Coulter, CA, USA) were used to choose suitable fragments. The sequencing library was enriched with PCR amplification. After verification with Qubit 2.0 and quantification with Agilent 2100, the effective concentration was accurately quantified using qRT-PCR. The library was then sequenced with an Illumina HiSeq2500 platform (Biomarker Technology Co., Ltd., Beijing, China).

### 4.3. Preprocessing of Illumina Reads

The cDNA library of each sample was sequenced by Illumina HiSeq2500 based on sequencing by synthesis technology. This library produced amounts of high-quality reads (most bases reached or had excess Q30). Preprocessing of reads involved Q20-quality trimming (removal of low-quality reads with an average Phred quality score of 20, trimming of low-quality bases from the 3′ ends of the reads) and removal of adapter/primer contamination and duplicated reads using custom Perl scripts. Preprocessed reads were sorted into individual samples based on barcodes using fastx_barcode_splitter. Barcodes were trimmed using fastx_trimmer from Fastx_toolkit.

Prior to assembly, the raw reads were cleaned by removing adapter sequences using standard Illumina pipeline, namely, the CASSAVA program (http://support.illumina.com/sequencing/sequencing_software/casava.ilmn). Adaptor contamination was removed, and reads were screened from the 3′ to 5′ ends to trim the bases with a quality score of *Q* < 20 using 5 bp windows. Reads with final length < 25 bp were removed.

### 4.4. De Novo Transcriptome Assembly

The Trinity software package (http://trinityrnaseq.github.io/) was used for de novo assembly of *P. aegyptiaca* transcriptome from preprocessed RNA-seq reads [32]. A K-mer library was built using the sequencing reads, and K-mer, which probably contained errors, was deleted. The highest frequency of K-mer was used as seed to extend greedily to both ends. This process was continuously cycled until the K-mer ran out. The contigs were clustered to obtain components. A simplified De Bruijn diagram of each contig of each component was constructed. Finally, the graph was unlocked with true reads to obtain transcript sequences. Trinity combines reads of a certain length of overlap to form longer fragments and then processes them for sequencing. The resultant sequences were defined as unigenes for downstream analysis.

### 4.5. Functional Annotation and Analysis

The assembled contig sets were compared against various databases (NCBINR, Swissprot, GO, COG, KOG, and KEGG databases) using BLASTX (http://blast.ncbi.nlm.nih.gov/Blast.cgi) with an E-value threshold of 1 × 10^−5^ [80]. Predicted amino acid sequences of unigenes were searched against the Pfam [81] database using HMMER software (http://hmmer.org/) with an *E*-value ≤ 1 × 10^−10^ to gain the unigene annotation information.

GO is a controlled vocabulary of terms split into three related ontologies consisting of molecular function, biological processes, and cellular components. It has a hierarchical structure that forms a directed acyclic graph in which each term has defined relationships to one or more other terms in the same domain. This characteristic can be described as a parent–child relationship. Each GO term is represented by a node in this graph, and the nodes are annotated with a set of genes. GO annotation was performed from gene expression data using TopGO [82].

The KEGG pathways were assigned to the assembled unigenes using the KAAS server automatic annotator to summarize the pathway information involved in *P. aegyptiaca* seed germination [83]. The transcripts were aligned to sequences in the COG database (http://www.ncbi.nlm.nih.gov/COG) and categorized under different functions accordingly.

### 4.6. Differential Expression Analysis

EBseq is a widely accepted and accurate analysis of RNA-seq data [84]. This process was performed to identify DEGs. RPKM values were used to measure gene expression levels. FDR was used to determine the threshold of the *p*-value in multiple tests [85]. FDR < 0.01 and fold change ≥2 were considered the cutoff threshold to determine the significance of expression. Cluster of significantly regulated genes was assessed with Cluster [86], MeV [87] and TreeView [88].

### 4.7. qRT-PCR Validation

Total RNA for RNA-seq samples was also used for qRT-PCR analysis. First-strand cDNA was synthesized using M-MLV reverse transcriptase (Roche, Switzerland) and oligo (dT) 18. We chose 8 plant hormone-related genes, 3 SL perception-related genes and randomly selected 12 genes for further study and validation of RNA-seq using qRT-PCR. Each transcript primer (Table S1) was designed using Primer Premier 5.0 (Premier Biosoft International, Palo Alto, CA, USA). OaTublin1 was used as an internal control [89]. qRT-PCR was conducted using SYBR GreenER™ qPCRSuperMix Universal (Invitrogen, Gaithersburg, MD, USA) according to the manufacturer’s instructions. Thermal cycle conditions for PCR were as follows: 94 °C for 3 min, 40 cycles including 94 °C for 15 s and 60 °C for 30 s. Relative expression levels were calculated using the 2^−ΔΔ*C*t^ method [90] and one-way ANOVA (IBM SPSS Statistics 19.0, Armonk, NY, USA).

### 4.8. Endogenous Hormone Level Analysis

Approximately 1 g (weighed after 5 min of air-drying) of seeds for each sample (for RNA-seq) was collected. Fresh material was frozen in liquid nitrogen and ground to a fine powder. ABA, IAA, and GAs were extracted according to the method of Durgbanshi with slight modification [91]. BRs were extracted using the method of Kim [92]. The four plant hormones were quantified using appropriate ELISA kits (Chengling, Beijing, China) according to the manufacturer’s instructions [93]. The released ethylene level was quantified according to the method of Cristescu [94]. All quantifications were performed with triplicate samples of each treatment, and data were analyzed by one-way ANOVA (IBM SPSS Statistics 19.0, Armonk, NY, USA).

### 4.9. Seed Hormone Treatments

*P. aegyptiaca* seeds were prepared as previously described. IAA (0.1% ethanol, *v*/*v*), GA3 (0.1% ethanol, *v*/*v*), epi-brassinolide (0.1% ethanol, *v*/*v*), ethephon, ABA (0.1% ethanol, *v*/*v*), fluridone (0.1% acetone, *v*/*v*) (inhibitors of carotenoid biosynthesis), or distilled water (as a control) was added to each Petri dish.

Each hormone, inhibitor, or water was added before conditioning in the first set of treatments. Hormones, inhibitors, or water were applied after 7 days of conditioning as the second group of treatments. Germination percentage was examined under a binocular microscope (Zeiss, Oberkochen, Germany) 3 days after GR24 treatment. Each treatment was performed with three replicate Petri dishes. Data were analyzed by one-way ANOVA (IBM SPSS Statistics 19.0, Armonk, NY, USA).

### 4.10. Accession Numbers

The quality filtered and trimmed short read data set, which was used in this study, was deposited to the NCBI Short Read Archive (SRA) under accession numbers: SRR2477710, SRR2477711, and SRR2477712.

## 5. Conclusions

This study generated a well-annotated transcriptome for the root parasitic weed, *P. aegyptiaca*. The taxonomy of the transcripts of this plant was highly similar to that of *S. indicum*. Thus, as expected, the parasite belongs to the same family of *S. indicum*. We clarified DNA repair and replication, RNA biosynthesis, and energy metabolism, as well as the transcription, translation, and biosynthesis of proteins, in this plant. We also found several key genes involved in seed germination, and these genes were associated with changes in the plant hormones. These genes may be developed as new targets to control *P. aegyptiaca*. These genes were validated by q-PCR and hormones levels were verified in seed germination experiments. Fluridone alone induced *P. aegyptiaca* seed germination, whereas ABA and ethylene were inferred as the key phytohormones during *P. aegyptiaca* seed germination. Our results may provide a perspective for suicidal germination to control parasitic weeds. In addition, we found that the expression levels of MAX2 and a KAI2 gene, which were involved in SL signal transduction, were always stable during the whole germination process. Thus, we deduced that transcripts of partial components of the SL perception complex existed prior to conditioning.

## Figures and Tables

**Table 1:** Assembly statistics for *Phelipanche aegyptiaca* transcriptome.

Length Range	Contig	Transcript	Unigene
Total number	11,469,131	274,964	94,419
Total length (bp)	670,369,345	293,096,156	66,808,988
N50 (bp)	53	1558	836
Average (bp)	58.45	1065.94	707.58

**Table 2:** *P. aegyptiaca* seed germination rate under different conditions.

Treatment	Germination (%)		
	No Conditioned	Conditioned	Conditioned and GR24 Treatment
BR	0 a	0 a	83.3 ± 3.8 fg
GA3	0 a	2.2 ± 2.2 a	94.4 ± 1.1 h
ABA	0 a	0 a	0 a
Ethephon	0 a	0 a	90.0 ± 1.9 gh
Fluridone	26.5 ± 1.8 b	7.8 ± 2.9 a	86.7 ± 5.7 gh
GA3 + Fluridone	35.6 ± 1.1 c	10.0 ± 3.3 a	91.1 ± 2.2 gh
Ethephon + Fluridone	24.4 ± 2.2 b	7.8 ± 1.1 a	80.0 ± 5.0 f
GA3 + Ethephon + Fluridone	47.8 ± 2.9 e	20.0 ± 1.9 b	91.1 ± 4.0 gh
Water	0 a	0 a	75.6 ± 9.1 f

“No conditioned” means that the dormant seeds were treated for 9 days directly without conditioning. “Conditioned” represents seeds conditioned in water at 25 °C in the dark for 7 days and then treated with plant hormones or inhibitor for 2 days. “Conditioned and GR24 treatment” involved seeds conditioned in water at 25 °C in the dark for 7 days and then treated with GR24 and other plant hormones or inhibitor for 2 days. The concentrations of hormones or inhibitor were as follows: BR, 1 mg/L; GA3, 10 mg/L; ABA, 1 mg/L; fluridone, 100 mg/L; ethephon, 500 mg/L at pH 5; and GR24, 10^−6^ mol/L, all the hormones were dissolved in 0.1% ethanol (*v*/*v*), except GR24 and fluridone were dissolved in 0.1% acetone (*v*/*v*). Means within a column followed by the same letter do not differ significantly according to Fisher’s protected LSD test (*p* < 0.05).

## Supplementary Materials

Supplementary materials can be found at http://www.mdpi.com/1422-0067/17/7/1139/s1.
