# Supplementary material for: Global Transcriptomic Analysis Reveals the Mechanism of Phelipanche aegyptiaca Seed Germination
Source: Int J Mol Sci. 2016 Jul 15;17(7):1139. doi: 10.3390/ijms17071139 (PMC4964512; doi:10.3390/ijms17071139)
Supplement: Supplementary file 1 [file ijms-17-01139-s001.pdf]

Supplementary Materials: Global Transcriptomic Analysis Reveals the Mechanism of *Phelipanche Aegyptiaca* Seed Germination

Zhaoqun Yao, Fang Tian, Xiaolei Cao, Ying Xu, Meixiu Chen, Benchun Xiang and Sifeng Zhao

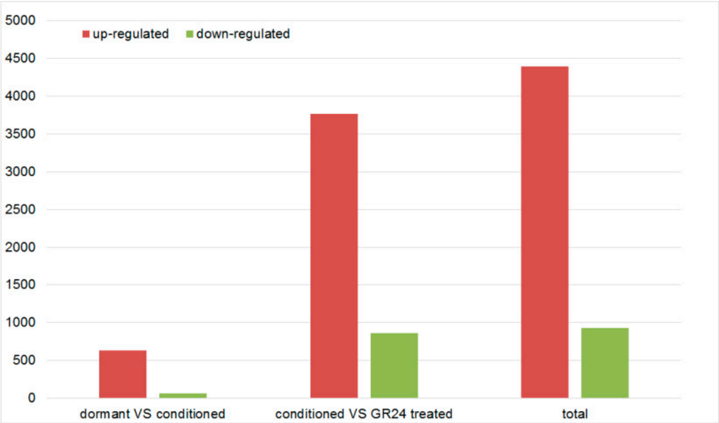

Figure S1. The number of up-regulatedand down-regulatedgenes among the three germination stages.

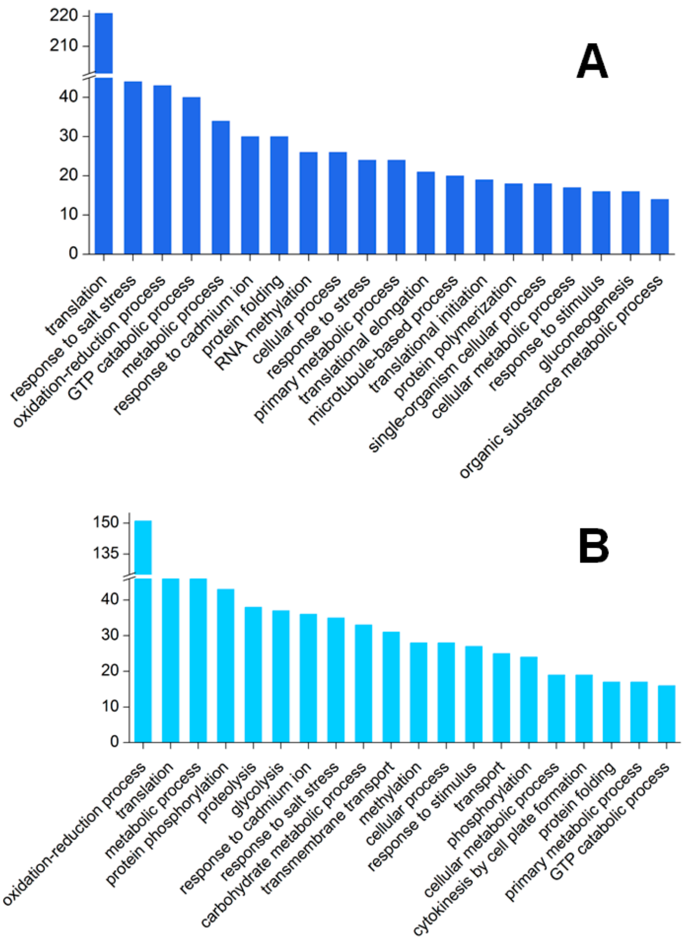

Figure S2. The top 20 GO terms enriched from dormant vs. conditioned (**A**) and conditioned vs. GR24 treated (**B**) DEGs.

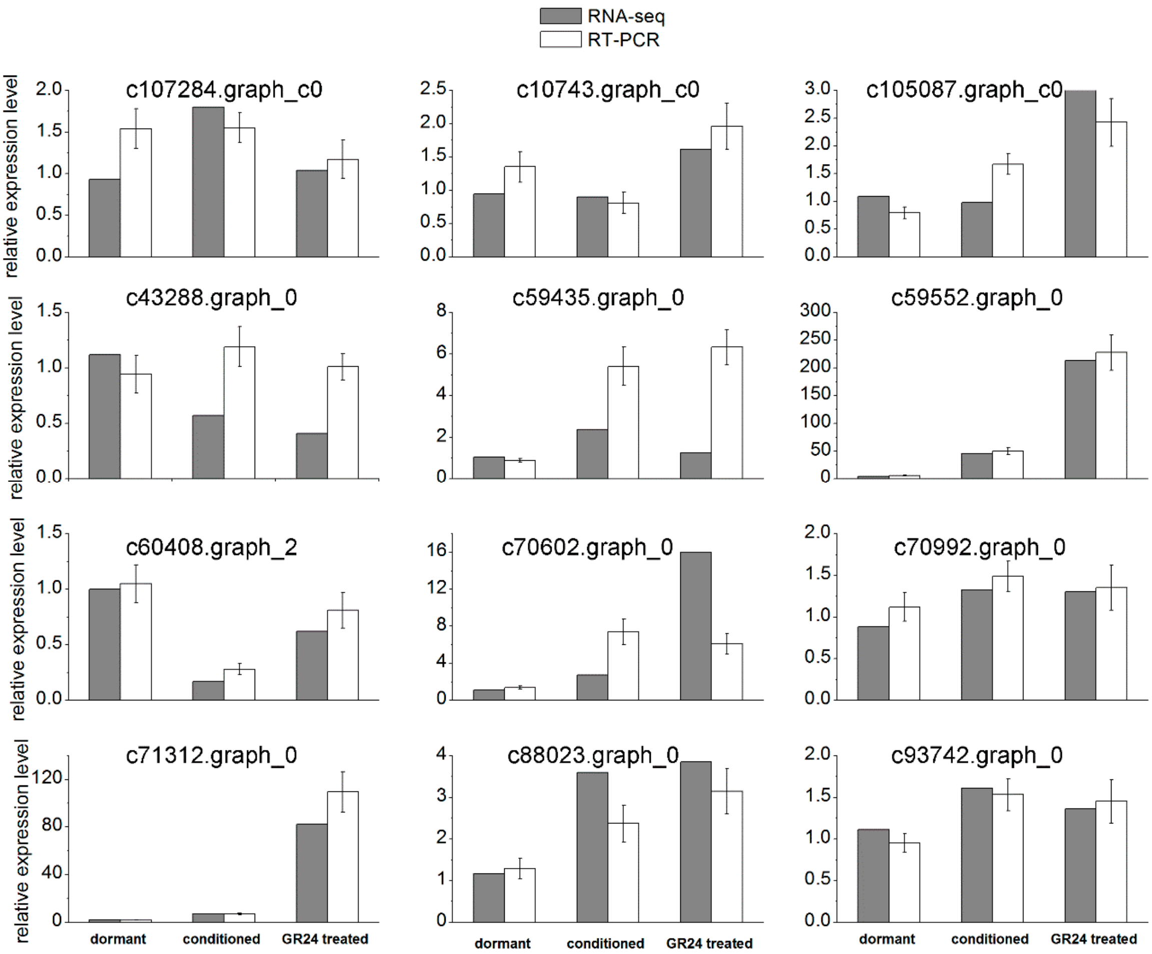

Figure S3. Comparison of expression profiles of 12 randomly selected genes by RNA-Seq and RT-PCR showing different expression profiles during three development stages. In all cases the results are the average of three biological replicates. The expression pattern results were consistent between qRT-PCR and RNA-seq analysis.

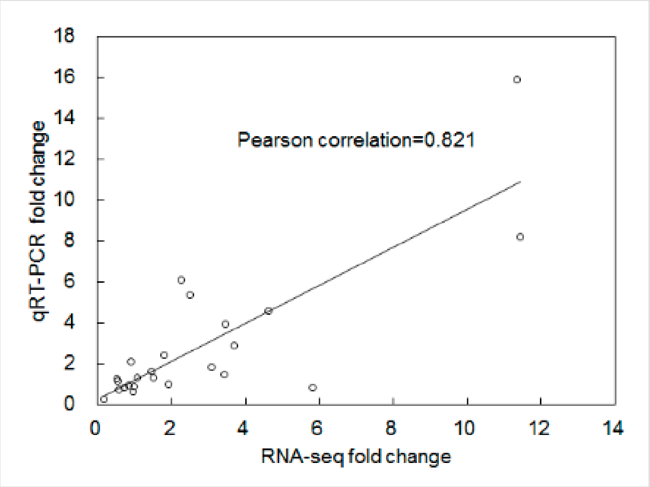

Figure S4. Correlation between RNA-seq and RT-qPCR data.Expression levels of the 12 randomly selected genes and hormones related genes were used for Pearson correlation analysis.

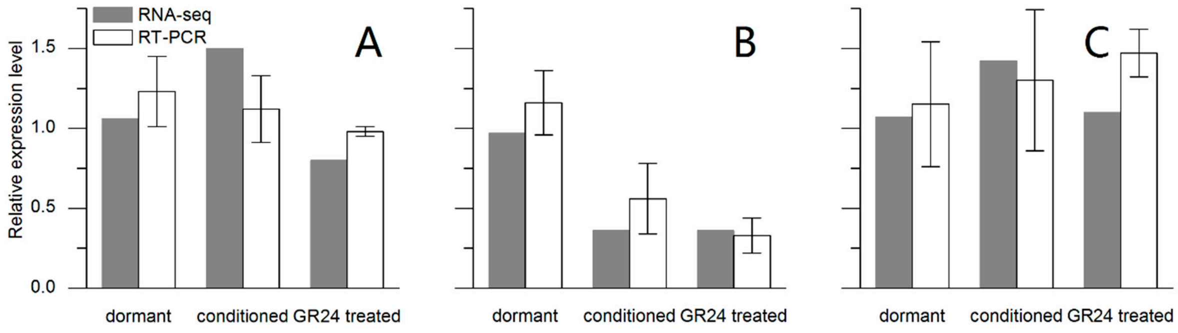

Figure S5. Validation of SLs receptor related genes during the seed germination stage by qRT-PCR. Fold changes were originally estimated in the RNA-Seq experiment (grey bar) and validated by qRT-PCR (white bar). The transcripts accumulation of *PaKAI2-like1*, *PaKAI2-like2* and *PaMAX2* were shown in **A**–**C**, respectively, at different germination stages. In all cases the results are the average of three biological replicates.

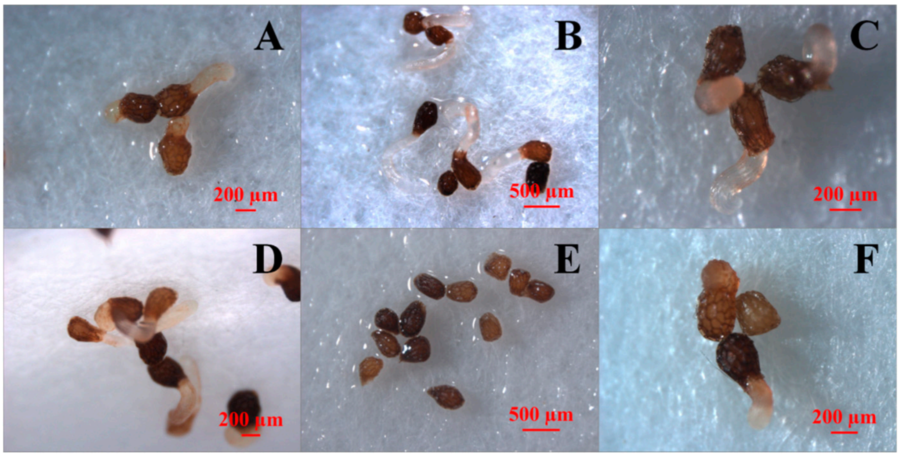

Figure S6. Pictures of *P. aegyptiaca*seeds germination under (**A**) Fluridone (100 mg/L); (**B**) GA3 (1 mg/L) + Fluridone (100 mg/L); (**C**) Ethephon (500 mg/L, pH 5) + Fluridone (100 mg/L); (**D**) GA3 (1 mg/L) + Ethephon (100 mg/L) + Fluridone (100 mg/L); (**E**) water without GR24 treatment; (**F**) GR24 treatment. All the agents were added at the beginning of conditioning stage, except GR24 was added after conditioning treatment.

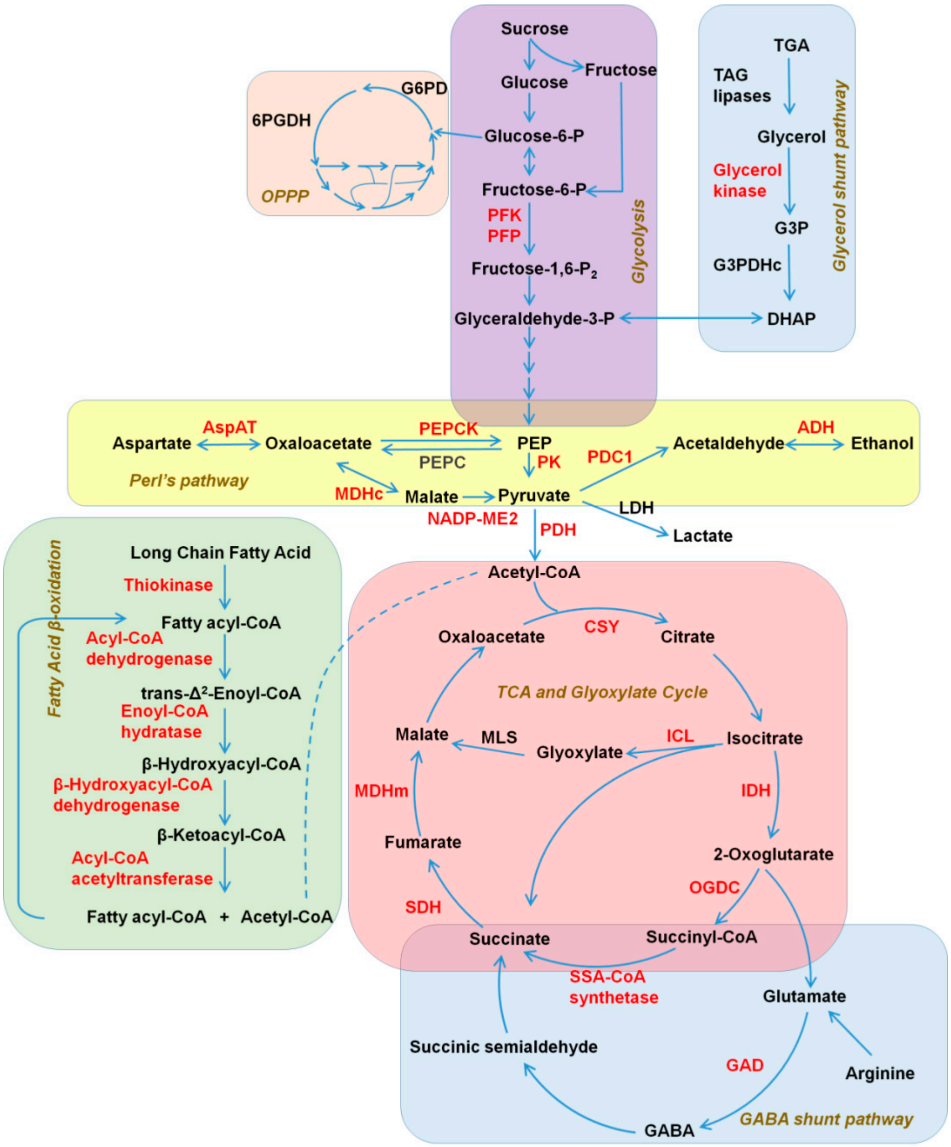

Figure S7. Reactivation of metabolic pathways and energy production during seed germination in *P. aegyptiaca*. Glycolysis, aerobic (TCA cycle), and anaerobic (fermentation) respiration are the commonly used pathways for ATP production. Transcript expression patterns for key genes representing these metabolic pathways are provided. Transcript levels are only presented if up-regulation was at least twofold. Key enzymes for which transcript levels are up-regulated are labeled in red, whereas others are labeled in black. Fermentation-related transcripts [pyruvate dehydrogenase complex (PDC) and alcohol dehydrogenase (ADH) but not lactate dehydrogenase (LDH)] were up-regulated during seed germination. The transcript expression patterns support the up-regulation of glycolysis [phosphofructokinase (PFK) (irreversible step), pyrophosphate-dependent phosphofructokinase (PFP), and pyruvate kinase (PK)], but not of gluconeogenesis (e.g., phosphoenol pyruvate carboxykinase (PEPCK)). Fatty acid β-oxidation, glyoxylate cycle (isocitrate lyase (ICL)), and the γ-aminobutyric acid (GABA) shunt pathway (glutamate decarboxylase (GAD)) were up-regulated. The TCA cycle (citrate synthase (CSY), isocitrate dehydrogenase (IDH), oxoglutarate dehydrogenase complex (OGDC), succinyl-CoA ligase (SCoS), succinate dehydrogenase (SDH), and malate dehydrogenase (MDH)) were active in *P. aegyptiaca* seed germination. Seed-specific routes that may contribute to ATP production were as follows: the glycerol shunt pathway in which glycerol kinase was up-regulated, but not TAG lipases and glycerol-3-phosphate dehydrogenase (G3PDHc)and (2) Perl's pathway (PEP carboxylase (PEPC), MDHc, and PK) which includes amino acid aminotransferases (AspAT and AlaAT).

Table S1. Primers used in qRT-PCR analysis.

Name		Sequence (5′ to 3′)
PaGA2OX1	Sense	CTCGCTGAAAGATGGCAACTGG
	Anti-sense	AACGGTGGCCCTCCGAAATA
PaGA3OX1	Sense	AGCTCATGTGGTCCGAAGGGTT
	Anti-sense	GGGCTTTCATTGCGTTCTGGTA
PaNCED	Sense	CGACCCATAATCCCACCCTCA
	Anti-sense	TGCCAGAACATACCCATCGTCC
PaCYP707A1	Sense	TCCATGCCCATCAATCTTCCC
	Anti-sense	TCCATGAACGAGCCCAGCAA
PaMAT	Sense	ATGGCGATGCGGGTTTGACT
	Anti-sense	CTTAGCCGCCTGCCTCACAATA
PaACS	Sense	GGACCTCAAGTGGCGAACCG
	Anti-sense	CCCAGGGGATTGGAGGGATTA
PaACO	Sense	ATACGAATGCCACCGCCTTGT
	Anti-sense	GACCCACGCCCCGTCTTTTA
PaCYP90B1	Sense	AAGGGAACAGGGGAGACGGC
	Anti-sense	TGGCAACACTTTCCACCCACAT
Patublin1	Sense	GGTCCCGAAAGATGTCAACGC
	Anti-sense	GAGAACACCTCCGCCACGCT
c107284.graph_c0	Sense	TCTCCATCAGTTTCATGATTATGCT
	Anti-Sense	CCCTAACATTGGTCAAAATCCC
c10743.graph_c0	Sense	AGGATCGTGCTAACCCGATATG
	Anti-Sense	TCGGAGTGGAGGGAGCAAAT
c105087.graph_c0	Sense	AATAACAAACCAGCGCATCGA
	Anti-Sense	GCCGTAGCACAAAATAATTGCA
c43288.graph_c0	Sense	AGAACTCAAGCCGAATGTCTCC
	Anti-Sense	GCCCTCGGCAGGAACAGA
c59435.graph_c0	Sense	ACGCTGTTACCGCCTCCAG
	Anti-Sense	ACGGCCCTCAGACTCCTCC
c59552.graph_c0	Sense	CATCAGCAGCGGCAACAGC
	Anti-Sense	GCATCAATGGTCGCAAATCC
c60408.graph_c2	Sense	TGGATGAAGTGAGATTGGAGCA
	Anti-Sense	TATCAGAGTTGTCGCCATGAGC
c70602.graph_c0	Sense	CGTAACTCGGCTTTGACCCA
	Anti-Sense	CCGCCTCAACGGTTTCTGT
c70922.graph_c0	Sense	GACATTTATTGCTGCCGGTTTG
	Anti-Sense	GTAACTGCTGAGTGAAAATTGCCAA
c71312.graph_c0	Sense	GAACAAGTGGAATTGGATGGCC
	Anti-Sense	CGCAGTTGGATGCTTACAAAGA
c88023.graph_c0	Sense	ACTGCCGAAATTAAACTCACCC
	Anti-Sense	TGAAGATGGCAAACACCGTTC
c93742.graph_c0	Sense	CTTGAAGGAGGAAGGGGATTTG
	Anti-Sense	TGTAGACACGGCGTTGGTGC
KAI2-like1	Sense	GCCACGCTGAGGGCTATGT
	Anti-Sense	TGAACGCGATGGAAACGAAC
KAI2-like2	Sense	TTTCTGAGTATCGCCACCAAGG
	Anti-Sense	AGGTCACGTCACTGTCCCATG
MAX2	Sense	CGCCCTCATCTCAGTGCTCA
	Anti-Sense	GCGACATCCTCGGGTTCG
